# Decrease in Plasma Levels of α-Synuclein Is Evident in Patients with Parkinson’s Disease after Elimination of Heterophilic Antibody Interference

**DOI:** 10.1371/journal.pone.0123162

**Published:** 2015-04-07

**Authors:** Ryotaro Ishii, Takahiko Tokuda, Harutsugu Tatebe, Takuma Ohmichi, Takashi Kasai, Masanori Nakagawa, Toshiki Mizuno, Omar M. A. El-Agnaf

**Affiliations:** 1 Department of Neurology, Kyoto Prefectural University of Medicine, Kyoto, Japan; 2 Department of Molecular Pathobiology of Brain Diseases, Kyoto Prefectural University of Medicine, Kyoto, Japan; 3 Department of Zaitaku (Homecare), Kyoto Prefectural University of Medicine, Kyoto, Japan; 4 Department of Medical Education and Primary Care, Kyoto Prefectural University of Medicine, Kyoto, Japan; 5 North Medical Center, Kyoto Prefectural University of Medicine, Kyoto, Japan; 6 College of Science, Engineering and Technology, HBKU, Education City, Qatar Foundation, Doha, Qatar; Louisiana State University Health Sciences Center, UNITED STATES

## Abstract

There is substantial biochemical, pathological, and genetic evidence that α-synuclein (A-syn) is a principal molecule in the pathogenesis of Parkinson disease (PD). We previously reported that total A-syn levels in cerebrospinal fluid (CSF), measured with the specific enzyme-linked immunosorbent assay (ELISA) developed by ourselves, were decreased in patients with PD, and suggested the usefulness of A-syn in CSF and plasma as a biomarker for the diagnosis of PD. After our report, a considerable number of studies have investigated the levels A-syn in CSF and in blood, but have reported inconclusive results. Such discrepancies have often been attributed not only to the use of different antibodies in the ELISAs but also to interference from hemolysis. In this study we measured the levels of A-syn in CSF and plasma by using our own sandwich ELISA with or without heterophilic antibody (HA) inhibitor in 30 patients with PD and 58 age-matched controls. We thereby revealed that HA interfered with ELISA measurements of A-syn and are accordingly considered to be an important confounder in A-syn ELISAs. HA produced falsely exaggerated signals in A-syn ELISAs more prominently in plasma samples than in CSF samples. After elimination of HA interference, it was found that hemolysis did not have a significant effect on the signals obtained using our A-syn ELISA. Furthermore, plasma levels of A-syn were significantly lower in the PD group compared with the control group following elimination of HA interference with an HA inhibitor. Our results demonstrate that HA was a major confounder that should be controlled in A-syn ELISAs, and that plasma A-syn could be a useful biomarker for the diagnosis of PD if adequately quantified following elimination of HA interference.

## Introduction

Idiopathic Parkinson disease (PD) is the second most common neurodegenerative disorder after Alzheimer’s disease. It is pathologically characterized by the presence of Lewy bodies and Lewy neuritis in the substantia nigra and several other subcortical regions [1]. α-Synuclein (A-syn) is a major component of Lewy bodies and therefore may serve as a promising candidate biomarker for Parkinson’s disease (PD). We demonstrated that the concentration of A-syn in cerebrospinal fluid (CSF) in patients with PD is significantly decreased compared with that of age-matched controls using an originally developed enzyme-linked immunosorbent assay (ELISA) [2]. Since then, our result has been reproduced by many groups [3–10]. However, measurements of A-syn levels in CSF have varied among reports. Some researchers have reported that there are no differences in the CSF levels of A-syn between PD and controls [11–15]. Such inconsistencies have often been attributed to blood contamination causing hemolysis of red blood cells (RBCs) that contain abundant A-syn [5], while interference from heterophilic antibodies (HAs) has not been recognized as a confounder of ELISAs in this field. HAs are "human antibodies capable of binding to animal immunoglobulins and possibly of interfering with reaction of animal-derived antibodies and analyte, which comprise all immunoassays. The three groups of heterophilic antibodies are polyspecific antibodies, antiimmunoglobulin antibodies, and high-specificity high-affinity antibodies against antigens from animal species (http://www.medilexicon.com/medicaldictionary.php?t=4741)” [16–19]. HAs generally produce falsely exaggerated signals through cross-binding of capture and reporter antibodies used in ELISAs [17, 18] (S1(B) Fig). However, HAs can produce false-negative signals, though less frequently, when HAs occupy antigen binding sites of the capture and/or reporter antibodies [18] (S1(C) Fig). It was reported that HAs were found in up to 40% of human sera, and assay interference from HA occurs in as many as 15% of serum samples despite highly depending on the specific assay setup [17, 19]. HA interference does not need to be considered in immunoassays when there is a large amount of target antigen that provides strong signals [17]. However, the presence of HAs cannot be ignored in micro-quantitative ELISAs, because non-specific HA binding can often affect the delicate interaction between target antigens and antibodies employed in the ELISA [17]. Therefore, eliminating interference from HAs must always be considered in cases where immunoassays are used to measure small amounts of proteins [20]. With respect to A-syn immunoassays, the role of HA interference in the various A-syn ELISAs previously reported has not been investigated, although these ELISAs were aimed at measuring very small quantities of A-syn in human body fluids. To address this issue, this study first aimed to clarify which is the major confounder in our A-syn ELISA system, HAs or hemolysis (blood contamination).

Blood-based tests are less invasive and more suitable for mass screening than CSF-based tests. In fact, a considerable number of reports assessing A-syn concentrations in blood have been published to date [6, 13, 21–27]. However, the results of those reports were highly inconsistent. We assume that the poor reproducibility was partly due to A-syn derived from RBCs, as previously described [28], but also largely due to interference from HAs not being taken into account in previous studies. In general, HAs interrupt assays of blood samples more compared to CSF samples [19]. Therefore, if HAs have affected the reported A-syn ELISAs, cross-sectional studies that have quantified plasma and CSF A-syn concentrations in PD or other neurological diseases should be re-examined using accurate ELISAs in which the effect of HAs is eliminated. There are some reagents that have been reported to block or reduce HA interference, such as nonimune animal serum, nonimmune globulin, polyclonal IgG, mixture of mouse monoclonal antibodies, and so on[16, 17]. The second purpose of this study was to accurately measure A-syn levels in CSF and plasma using a commercially available HA inhibitor (HAI), in order to evaluate the usefulness of CSF and plasma A-syn levels as PD biomarkers, as well as to examine the relationship between A-syn levels in CSF and plasma.

## Materials and Methods

### Ethics Statement, Subjects Recruitment and sample collection

All study subjects were informed of the purpose of the study, the experimental procedures and all the potential risks involved, and then gave us written consent. The study was approved by the local Ethics Committee of Kyoto Prefectural University and conformed to standards for the use of human subjects in research as outlined in the current Declaration of Helsinki (http://www.wma.net/en/30publications/10policies/b3/index.html). The patients were registered in this institute from April 2009 to August 2013. In Study I, we compared the levels of A-syn between samples treated with and without HAI using the CSF or plasma samples. In Study II we quantified A-syn levels in CSF and plasma samples with HAI. In Study III, we quantified the levels of Hb in the CSF and plasma samples. In study II, we enrolled 30 patients with non-familial PD diagnosed according to the UK PD Society Brain Bank criteria (12 men / 18 women, aged 46 to 82, mean ± standard deviation (SD); 65.7 ± 9.7 yr) in the Department of Neurology, Kyoto prefectural University of Medicine. We also enrolled 58 participants as age-matched controls (22 men / 36 women, aged 30 to 79, mean ± SD; 59.6 ± 14.7 yr) in the same registration (Table 1). The control group in study II comprised neurologically normal individuals (n = 10) and patients with various neurological disorders including peripheral neuropathy (n = 20), cervical spondylosis (n = 10), myopathy/myositis (n = 10), myelopathy (n = 6), and epilepsy (n = 2). All the samples were taken when the participants were required to give CSF and blood for routine clinical diagnosis or treatment. Since some of the samples obtained from the subjects in study II had run out, we used remaining samples in study I and III, which consisted of those from 23 patients with PD (11 men / 12 women, aged 46 to 82, mean ± standard deviation (SD); 64.3 ± 9.8 yr) and 45 age-matched controls (16 men / 29 women, aged 30 to 79, mean ± SD; 59.0 ± 13.8 yr) (Table 1).

**Table 1:** Demographics of the subjects and clinical characteristics of the patients with Parkinson’s disease (PD).

		Study I and III^$^		Study II^#^	
		Control	PD	Control	PD
Number of cases		45	23	58	30
Sex (F/M)		16/29	11/12	22/36	12/18
Age (years)*	Mean ± SD	59.0 ± 13.8	64.3 ± 9.8	59.6 ± 14.2	65.7 ± 9.7
	Range	30–79	46–82	30–79	46–82
UPDRS motor score	Mean ± SD	-	22.7±10.8	-	22.1±9.9
	Range	-	5–47	-	5–47
Hoehn and Yahr scale	Mean ± SD	-	2.7±1.0	-	2.6±1.0
	Range	-	1–4	-	1–4
Duration of disease (years)	Mean ± SD	-	4.1±3.8	-	3.9±3.8
	Range	-	0.5–17.0	-	0.5–17.0

*The age of the control group was matched with the PD group (p = 0.09, Mann-Whitney statistic analysis).

^$^Study I compares the levels of A-syn in the CSF and plasma samples with or without HAI. Study III quantifies the levels of Hb in the CSF and plasma samples.

^#^Study II quantifies A-syn levels in CSF and plasma samples with HAI.

CSF samples were collected in polypropylene vials from PD and control cases in the morning (from 9 a.m. to 12 a.m.) through a lumbar puncture at the L3/L4 or L4/L5 interspace by referring to the Biologics Manual of the Parkinson’s Progression Markers Initiative biologics manual (http://www.ppmi-info.org/). Immediately after collection, the samples were cleared by centrifugation at 400 x g for 10 min at 4°C, and then a cocktail of protease inhibitors (Calbiochem-Novabiochem Corporation, San Diego, USA) was added to each sample. Blood was drawn through venipuncture into EDTA-containing collecting tubes soon after CSF collection, and plasma was separated by centrifugation at 2000 x g for 10 min at 4°C. CSF and plasma samples were aliquoted into polypropylene tubes, and stored at -80°C until used for ELISA.

### Measurement of A-syn with or without the elimination of HA interference

Total A-syn levels in CSF and plasma were measured using a sandwich ELISA system (211-FL140 ELISA) as previously described with some modification [4]. In brief, the anti-human A-syn monoclonal antibody 211 (Santa Cruz Biotechnology, CA, USA), which recognizes amino acid residues 121–125 of human A-syn, was used for antigen capturing. The anti-human A-syn polyclonal antibody FL-140 (Santa Cruz Biotechnology, CA, USA), raised against recombinant full-length human A-syn, was used for antigen detection through a horseradish peroxidase (HRP)-linked chemiluminescence assay. The ELISA plate (Nunc Maxisorb, NUNC, Denmark) was coated with 1 μg/ml of 211 (100 μl/well) in 200 mM NaHCO_3_ (Sigma–Aldrich, MO, USA), pH 9.6, containing 0.02% (w/v) sodium azide, washed four times with PBST (phosphate buffered saline (PBS) containing 0.05% Tween 20) and incubated with 200 μl/well of blocking buffer (PBS containing 2.5% gelatin and 0.05% Tween 20) for 2 hours. After washing with PBST, 100 μl of the samples diluted with or without HAI (ELISA diluent, MABTECH, Sweden) were added to each well and incubated at 37°C for 3 hours. Captured A-syn was detected using 0.2 μg/ml of FL-140 antibody (100 μl/well) diluted to 1:1000 in blocking buffer, followed by incubation with 100 μl/well (1:10,000 dilution) of HRP-labeled anti-rabbit antibody (DAKO, Denmark). Bound HRP activity was assayed by chemiluminescence using an enhanced chemiluminescent substrate (SuperSignal ELISA Femto Maximum Sensitivity, Thermo fisher scientific, MA, USA). Chemiluminescence in relative light units was measured with a microplate luminometer (SpectraMax Pro, Molecular Devices Corporation, Tokyo, Japan). The standard curve for the ELISA was carried out in each plate using 100 μl/well of recombinant human A-syn (rPeptide, GA, USA) solution at different protein concentrations in PBS. Relative concentration estimates of total A-syn in the samples were calculated according to the standard curve obtained in each plate. To eliminate inter-assay variability as a confounding factor, all measurements were conducted in triplicate (unless otherwise noted) and performed using the same lot of standards. Furthermore, we placed internal control samples in each plate to adjust plate-to-plate variability. The intra-assay and inter-assay variance was less than 10%.

### Measurement of hemoglobin levels

The hemoglobin (Hb) levels in CSF and plasma samples were measured using a Human hemoglobin ELISA Quantitation Kit from Bethyl Lab Inc (Montgomery, TX, USA) according to the manufacturer’s instructions.

### Blue native-PAGE and immunoblotting

Blue native-PAGE was performed using Novex 4–12% gradient gels (Thermo Fisher Scientific, MA, USA). The primary antibodies (C211 and FL-140) were prepared by addition of 5% Coomassie G-250 additive. Blue Native-polyacrylamide gels were then run at 150 V for 2 hours according to the manufacturer’s protocol, and the separated proteins were then transferred onto PVDF membranes (Merck Millipore, Germany). The membranes were then blocked with 5% nonfat milk in PBS containing 0.1% (v/v) Tween 20 for 30 min at RT, and subsequently incubated for 24 hours with plasma samples diluted to 50% in PBS, which exhibited either high or low HA activity in preparatory experiments. After washing with PBST, the membranes were incubated in HRP-labeled anti-human immunoglobulin antibody (DAKO, Denmark) for 3 hours. Immunosignals were visualized using chemiluminescence (ECL Select; GE Healthcare, England).

### Statistics

Mann-Whitney's U tests were used for comparisons between two independent groups. Correlation analysis was conducted using Spearman’s rank correlation coefficient test. The level of significance was set at p< 0.05. All analyses were performed with Graph Pad Prism for Windows (version 5.04, Graph Pad Software, Inc., CA, USA).

## Results

### Determination of appropriate HAI concentration for our A-syn ELISA

**Fig 1:**
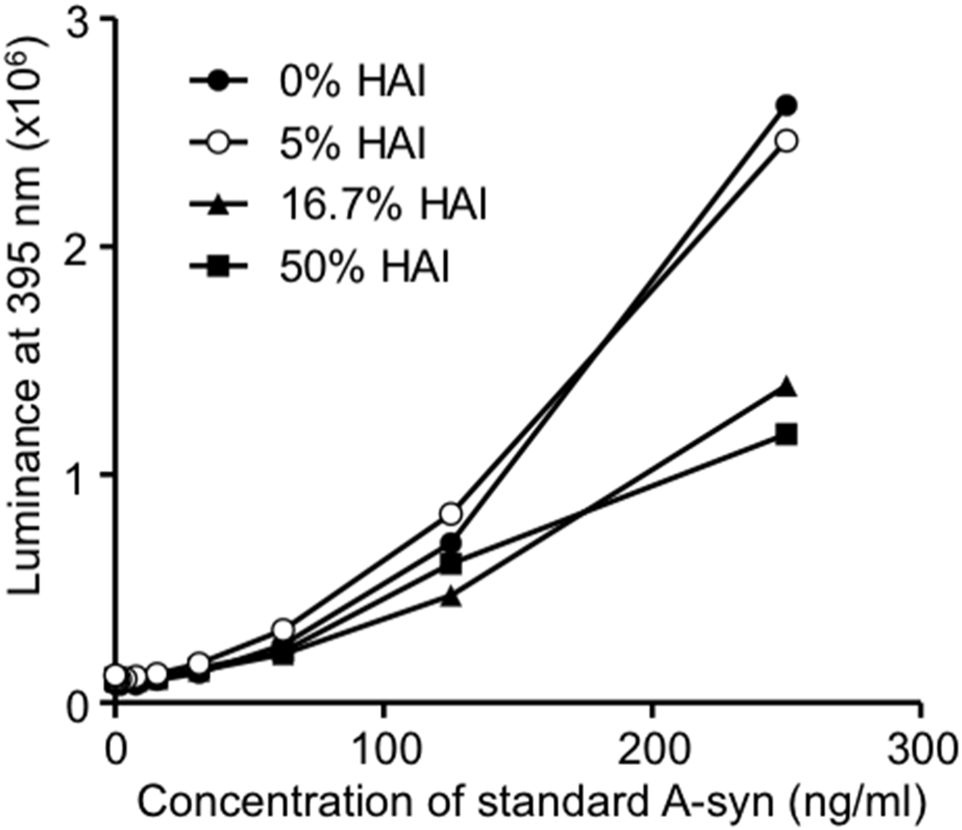
Determination of the optimal HAI concentration for the A-syn 211-FL140 ELISA. Chemiluminescence signals obtained from serially diluted recombinant A-syn in the presence of various HAI concentrations (0, 5, 16.7 and 50%) with the 211-FL140 ELISA. ELISA performance was not affected by 5% HAI, and was reduced in the presence of 16.7% and 50% HAI.

It is expected that optimal HAI concentration ranges will be specific to individual ELISA assay systems. To determine the appropriate HAI concentration for our A-syn ELISA (211-FL140 ELISA), we first performed an ELISA for recombinant A-syn (known concentration) diluted with various amounts of HAI. We found that use of high concentrations of HAI disrupted the ELISA performance (Fig 1), therefore a concentration of 5% HAI was selected for use in subsequent A-syn ELISA experiments, to preclude HA from falsely exaggerating ELISA signals and to avoid HAI disturbing ELISA performance.

### Effect of HAI pretreatment on A-syn ELISA signals

**Fig 2:**
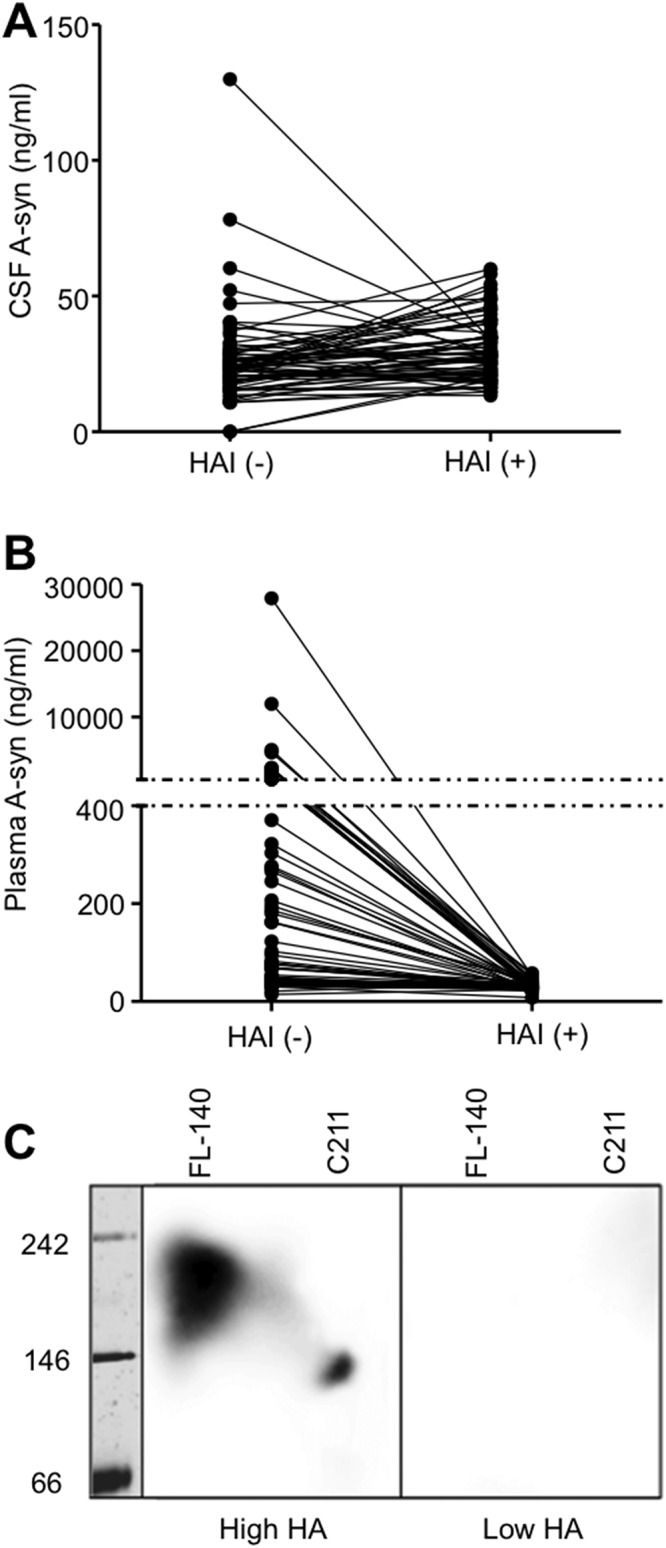
Effects of HA on measurements of CSF and plasma A-syn levels using the 211-FL140 ELISA. Levels of CSF-A-syn (A) and plasma-A-syn (B) were measured using the 211-FL140 ELISA in the absence and presence of 5% HAI. HAI pretreatment slightly increased A-syn signals in the majority of CSF samples (A), while the A-syn signals were remarkably decreased in most of the plasma samples (B). (C) In order to detect HA in plasma samples, the primary antibodies (C211 and FL-140) were separated by blue native-PAGE and immunoblotted with two plasma samples. The plasma sample on the left exhibited high HA activity, while the sample on the right had low HA activity in previous experiments that had determined the effects of HAI on the 211-FL140 ELISA. The plasma with high HA activity clearly reacted with both the 211 and FL-140 antibodies, while the plasma with low HA activity did not react with either.

We used 68 paired samples of CSF and plasma obtained from 23 patients with PD and 45 controls to determine whether pretreatment with HAI could alter signals in the 211-FL140 ELISA (Study I in Table 1). Note that we did not use all of the control samples due to insufficient quantities. In the CSF samples, pretreatment with HAI slightly increased the A-syn signals detected with the 211-FL140 ELISA in the majority of the samples (n = 48; 13 from PD patients, 35 from controls). On the other hand, the A-syn signals in some samples (n = 20; 10 PD, 10 controls) were decreased with HAI pretreatment. In four of the 68 CSF samples showing relatively high A-syn values (>50 ng/ml) in the absence of HAI, the A-syn signals were consistently decreased with HAI pretreatment (Fig 2A).

In the plasma samples, HAI-pretreatment decreased the signals of A-syn detected with the 211-FL140 ELISA in the majority of the samples (66 out of 68). The other two samples, where plasma A-syn levels were slightly increased with HAI pretreatment, had the lowest plasma A-syn levels in this cohort measured in the absence of HAI (Fig 2B).

### Blue native-PAGE and immunoblotting

We conducted blue native-PAGE to confirm that the signal reduction observed in the plasma samples was caused by HA. The antibodies employed in our A-syn ELISA (211 and FL140) were separated by native PAGE and analyzed with immunoblotting. We then performed immunoblotting using the plasma samples as the primary antibodies and anti-human immunoglobulin as the secondary antibody to detect HA against both the capture (211) and detection (FL140) antibodies of our ELISA. We chose two plasma samples, with either high or low HA activity in the experiments of 211-FL140 ELISAs with or without HAI. In the plasma sample with high HA activity, there were strong bands that reacted with the FL-140 and 211 antibodies (Fig 2C). Conversely, these signals were not detected in the plasma sample with low HA activity (Fig 2C). This result clearly demonstrated that the human plasma with a high HA activity contained autologous immunoglobulins that bind to both the capture and detection antibodies and could bridge those antibodies so as to produce falsely exaggerated signals. However, the plasma sample with low HA activity did not contain such autologous immunoglobulins.

### Comparison of CSF and plasma A-syn with elimination of HA interference

**Fig 3:**
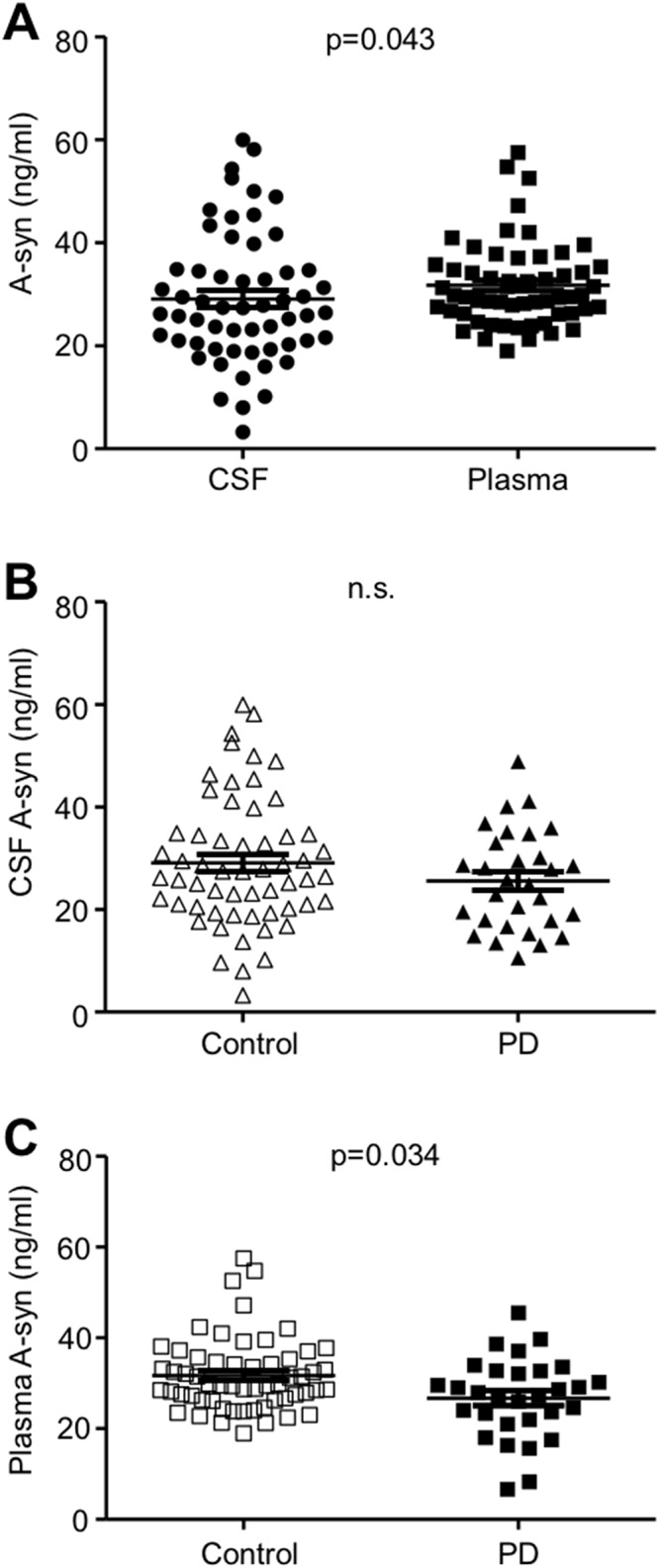
Comparison of A-syn levels measured using the 211-FL140 ELISA with HAI pretreatment. Values of A-syn obtained from individual subjects are plotted. (A) A-syn levels are compared between CSF and plasma in the overall samples. (B, C) The levels of CSF (B) and plasma (C) A-syn are compared between the control and PD groups. Long and short horizontal bars represent mean and standard deviation, respectively. The mean plasma A-syn levels was significantly higher than mean CSF A-syn (A; p = 0.043). The mean CSF and plasma A-syn levels were lower in the PD group compared to the control group (B, C), but a significant difference was only observed in the plasma samples (C; p = 0.034).

We measured the concentrations of A-syn in 88 CSF and plasma (PD: 30, control: 58) samples using the 211-FL140 ELISA with HAI pretreatment (Study II in Table 1). We compared A-syn concentrations between CSF and plasma as well as between PD and controls. In the overall samples combined, the mean value of the plasma A-syn levels was significantly higher than that of CSF (p = 0.043) (Fig 3A). In comparing the levels of CSF A-syn between the PD and control groups, the mean value of CSF A-syn levels was lower in the PD group than in the control group, however, the difference did not reach the level of significance (p = 0.25) (Fig 3B). On the other hand, we found that the mean value of plasma A-syn levels was significantly decreased in the PD group compared to the control group (p = 0.03) (Fig 3C).

The levels of plasma A-syn showed a tendency to decrease with age, both in the PD (p = 0.03) and control groups (p = 0.10). Furthermore, the age of the PD patients and plasma A-syn levels were significantly correlated (S2 Fig). In CSF samples, there was no correlation between the age of the subjects and A-syn levels in the PD (p = 0.40) or control groups (p = 0.46) (S2 Fig).

### Correlation between A-syn and Hb levels

**Fig 4:**
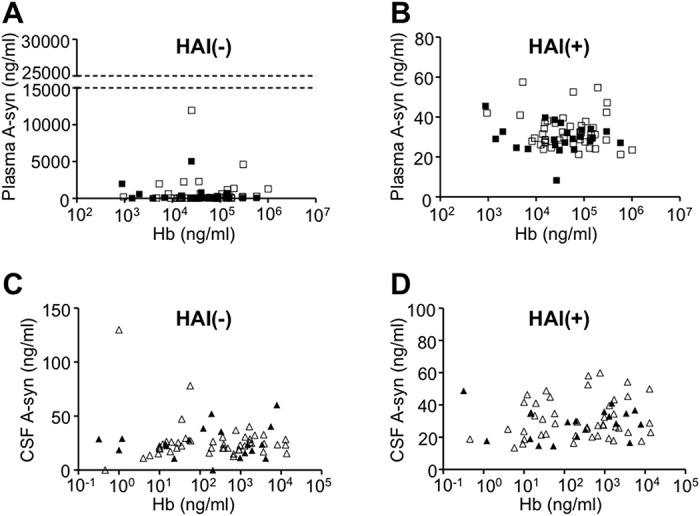
Correlations between Hb and A-syn levels in plasma and CSF. (A, B) Scatter plots of the levels of Hb versus those of A-syn in plasma measured without (A) or with (B) HAI pretreatment. (C, D) Scatter plots of Hb versus A-syn in CSF measured without (C) or with (D) HAI pretreatment. Closed rectangles and triangles indicate samples from the PD group, open rectangles and triangles are those from the control group. The horizontal axis is shown on a logarithmic scale. There were no significant correlations between the levels of Hb and plasma A-syn without (A; p = 0.86) or with (B; p = 0.39) HAI pretreatment. Significant correlations were not also observed between Hb and CSF A-syn without (C; p = 0.93) or with (D; p = 0.65) HAI pretreatment.

We measured the levels of Hb as a direct hemolytic marker in the samples (total 68: PD 23, control 45), and examined the correlation between the levels of Hb and A-syn measured using the 211-FL140 ELISA with or without the HAI pretreatment (Study III in Table 1). As shown in Fig 4, significant correlations were not identified between the plasma levels of Hb and A-syn without (A; p = 0.86) or with (B; p = 0.39) HAI pretreatment; neither were Hb and CSF A-syn without (C; p = 0.93) or with (D; p = 0.65) HAI pretreatment significantly correlated.

### Correlation between CSF and plasma A-syn levels with elimination of HA interference

**Fig 5:**
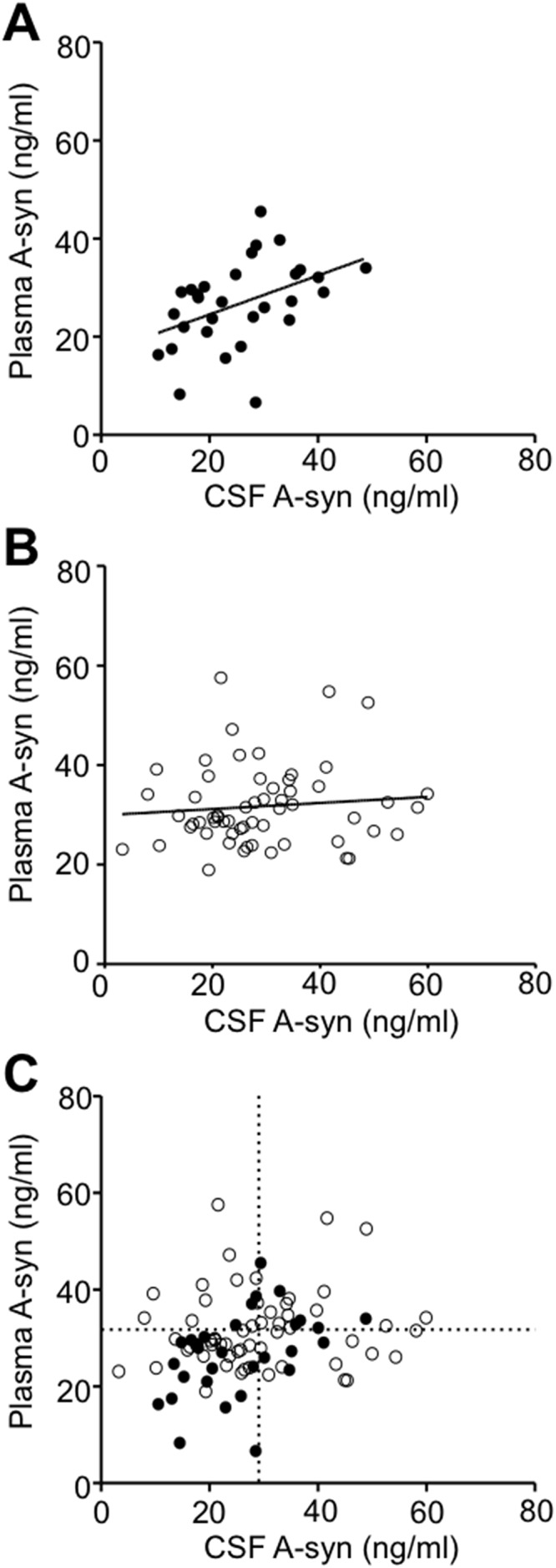
Correlations between A-syn levels in CSF and plasma samples obtained at the same time. Scatter plots of A-syn levels in CSF versus plasma in the PD group (A), control group (B), and the combination of PD patients and the controls (C). Closed and open circles indicate samples from the PD and the control groups, respectively. The solid lines indicate regression analyses. There was a significant positive correlation in the PD group (A) (p = 0.005). In the control group (B), no significant correlation was observed (p = 0.50). Dashed lines in (C) indicate the mean values of CSF (29.07 ng/ml) and plasma (31.72 ng/ml) A-syn in the controls, and are plotted to clarify the discrepancy between the PD and control groups with respect to the relationship between CSF and plasma A-syn levels. There was a tendency for patients whose CSF A-syn levels were lower than 29.07 ng/ml to exhibit plasma A-syn levels less than 31.72 ng/ml in the PD group (closed circles, Fig 5C); however, no such a tendency was observed in the control group (open circles, Fig 5C).

The levels of CSF and plasma A-syn, measured using the 211-FL140 ELISA with HAI pretreatment, were separately compared in the 30 PD and 58 control samples (Study II in Table 1). There was a significant positive correlation between the levels of CSF and plasma A-syn in the PD group (p = 0.005, Fig 5A), whereas no significant correlation was observed in the control group (p = 0.50, Fig 5B). To assist the explanation of the discrepancy between PD and control, lines were draw to indicate the mean values of CSF (29.07 ng/ml) and plasma A-syn (31.72 ng/ml) in the controls (dashed lines, Fig 5C). In the PD group (closed circles, Fig 5C), patients with levels of CSF A-syn lower than 29.07 ng/ml mostly had plasma A-syn levels lower than 31.72 ng/ml. This trend was not observed in the control group (open circles, Fig 5C). The PD subjects characterized by lower A-syn levels in both the CSF and plasma appeared to contribute the positive correlation between plasma and CSF A-syn which was only observed in the PD group, but not in the control group.

## Discussion

This study is the first report to demonstrate the interference of HAs in A-syn ELISA analysis as well as to quantify A-syn in human body fluids with elimination of HA interference.

First, we found that HAI pretreatment decreased signals in our A-syn ELISA in most of the plasma samples and a few CSF samples. Following elimination of HA interference, the levels of A-syn in all samples, both CSF and plasma, fell into a range of less than 60 ng/ml. Moreover, plasma samples exhibiting strong signal reductions following HAI pretreatment contained immunoglobulins capable of binding both the capture (211) and detection (FL140) antibodies employed in the A-syn ELISA. The HAI we used could prevent these immunoglobulins from binding the capture and reporter antibodies, despite detailed constituents of the HAI were not informed from the manufacture. These results suggest the following two hypotheses: 1) A-syn values greater than 50 ng/ml measured with the 211-FL140 A-syn ELISA without HAI pretreatment are likely to be falsely exaggerated by the presence of HAs in the examined samples; 2) HA interference is more prominent in plasma than in CSF. There have been two studies describing HA interference in Aβ ELISA. They found that HA generally affects micro-quantitative ELISA more strongly in plasma than in CSF, and produces false-positive rather than false-negative signals [19, 29]. Our results confirmed the presence of HA interference in the A-syn ELISA, and are consistent with previous reports. These findings suggest that HA is an important confounding factor that can generally affect ELISAs that measure very small amounts of antigens, and is not limited to the A-syn ELISA studied here. On the other hand, we also found that A-syn signals in some samples were slightly increased with HAI pretreatment. This phenomenon likely results from false-negative effects related to sample-derived HAs blocking antigen binding sites on either the capture or reporter antibodies. HAI can also eliminate such kind of HA interference in ELISA reactions. Another possible factor may be the presence of endogenous anti-A-syn antibodies in human plasma and CSF [30–33]. These autoantibodies could conceal the A-syn epitopes from the capture or reporter antibodies of the ELISA, thereby acting as a potential negative confounder in the A-syn ELISA without HAIs. HAI pretreatment might increase ELISA signals by blocking the negative effects of anti-A-syn autoantibodies.

Second, we also found that plasma A-syn levels with HAI pretreatment were significantly lower in the PD group than in the control group. Previous studies that quantified A-syn in plasma to elucidate its usefulness as a blood-based biomarker for the diagnosis of PD have lacked reproducibility [6, 13, 21–27], although none of those studies were adjusted for HA interference. Our results suggest that plasma A-syn could be a useful biomarker for the diagnosis of PD, and that using HAI pretreatment to eliminate HA interference in ELISA analysis is indispensable. On the other hand, A-syn levels in CSF of the PD group is lower than those of the control group, but the difference was not significant. Most case-control studies, including ours, reporting A-syn levels in CSF have demonstrated that there is considerable overlap between PD and control groups, with some reports failing to demonstrate significant differences [11, 12].

Third, we did not find a significant relationship between the levels of Hb and CSF or plasma A-syn. In contrast, previous studies have reported that hemolysis is a confounding factor that provides a strong positive signal in A-syn ELISAs [5, 34], because greater than 99% of A-syn in blood resides in red blood cells [28]. Those reports showed a weak but significant correlation between the CSF levels of A-syn and Hb only among samples with high levels of Hb [5, 34]. However, a considerable number of samples with high levels of Hb in those studies showed average or less than average levels of CSF A-syn [5]. These reports suggest that hemolysis does not necessarily produce an excessive A-syn signal in ELISAs. Another possibility is that our A-syn ELISA is less susceptible to hemolysis than those used in previous studies. Foulds et al. reported that the 211-FL140 A-syn ELISA is not easily affected by hemolysis [15]. Considering these facts, we conclude that HA interference, rather than contamination with red blood cells and hemolysis, is a major confounder in some ELISAs, just as in our 211-FL140 A-syn ELISA.

Plasma A-syn was slightly higher than CSF A-syn, even with elimination of HA interference in this study. This result is in agreement with the report of Mollenhauer et al. [6], despite their not using HAIs. In previous reports, A-syn levels in plasma were found to be 5–10 times higher than those in CSF [6], although there was substantial overlap between the ranges of plasma and CSF A-syn. Such a discrepancy can be attributable not only to the differences in the ELISAs employed in those studies [35] but also to the critical difference of whether or not HA interference was eliminated.

We found that the levels of CSF A-syn were positively correlated with the levels of plasma A-syn in the PD group, but not in the control group. This observed difference between the PD and control groups is likely due to the presence of subjects in the PD group, but not the control group, who were characterized by lower A-syn levels in both CSF and plasma (Fig 5C). Because of this tendency in the PD group, a positive correlation could be observed between the levels of CSF and plasma A-syn only in the PD group. Decreased CSF A-syn levels in PD are assumed to be due to intracellular aggregation and subsequent accumulation within affected neurons [2]. Accordingly, decreased A-syn levels both in CSF and plasma are thought to be attributable to A-syn deposition in systemic organs, as reported in adrenal gland [36], heart [37], gastrointestinal tract [38], and cutaneous autonomic nerves [39].

In conclusion, the present study indicates that the presence of HA is a major confounder in some ELISAs, including our A-syn ELISA. HA interference was more prominent in plasma than in CSF. Upon elimination of HA interference in the plasma, plasma A-syn levels were significantly lower in the PD group than in the control group. Moreover, after HA interference in the plasma was eliminated the plasma A-syn levels significantly correlated with the CSF A-syn levels in the PD group. These results indicate that plasma A-syn could be useful as a blood-based biomarker for the diagnosis of PD when adequately quantified by eliminating the interference of HAs.

## Supporting Information

S1 Fig Schematic illustration of HA interference. (A) Schema of how ELISAs detect the antigen (Ag), which is trapped between capture and reporter antibodies (Ab). (B) The form of HA interference in ELISAs which leads to a false positive result. The HA binds to both the capture and the reporter antibody simulating the presence of Ag in its absence. (C) The form of HA interference in ELISAs which leads to a false negative result. The HA binds to the capture (or the reporter) antibody and prevents antigen-antibody interaction.
(TIF)

S2 Fig Correlations between A-syn levels and the age of the subjects. (A, B) Scatter plots of the age of the subjects versus CSF A-syn levels in the control (A) and PD (B) groups. (C, D) Scatter plots of the age of the subjects versus plasma A-syn levels in the control (C) and PD (D) groups. The lines in the graphs represent regression lines, with solid and dashed lines representing significant and non-significant correlations, respectively. Regression analyses revealed a non-significant correlation between the age of the controls and CSF A-syn levels (A; p = 0.46) or plasma A-syn levels (C; p = 0.20), as well as between the age of the PD patients and CSF A-syn levels (B; p = 0.40). The correlation between the age of the PD patients and the levels of plasma A-syn was significant (D; p = 0.03).
(TIF)
