# Supplementary figures and images for: Decrease in Plasma Levels of α-Synuclein Is Evident in Patients with Parkinson’s Disease after Elimination of Heterophilic Antibody Interference
Source: PLoS One. 2015 Apr 7;10(4):e0123162. doi: 10.1371/journal.pone.0123162 (PMC4388641; doi:10.1371/journal.pone.0123162)

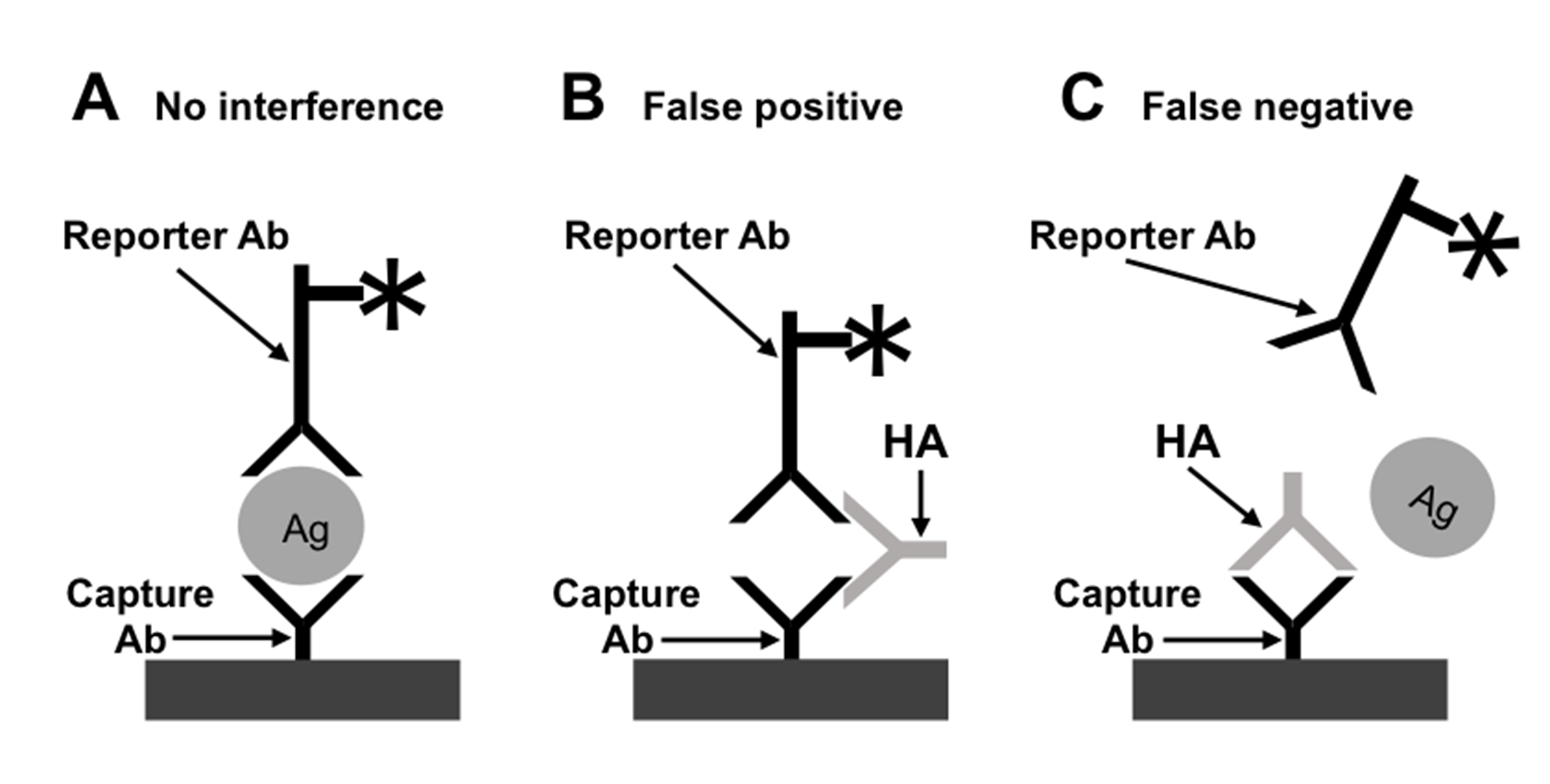

Supplement: S1 Fig — (A) Schema of how ELISAs detect the antigen (Ag), which is trapped between capture and reporter antibodies (Ab). (B) The form of HA interference in ELISAs which leads to a false positive result. The HA binds to both the capture and the reporter antibody simulating the presence of Ag in its absence. (C) The form of HA interference in ELISAs which leads to a false negative result. The HA binds to the capture (or the reporter) antibody and prevents antigen-antibody interaction. (TIF) [file pone.0123162.s001.tif]

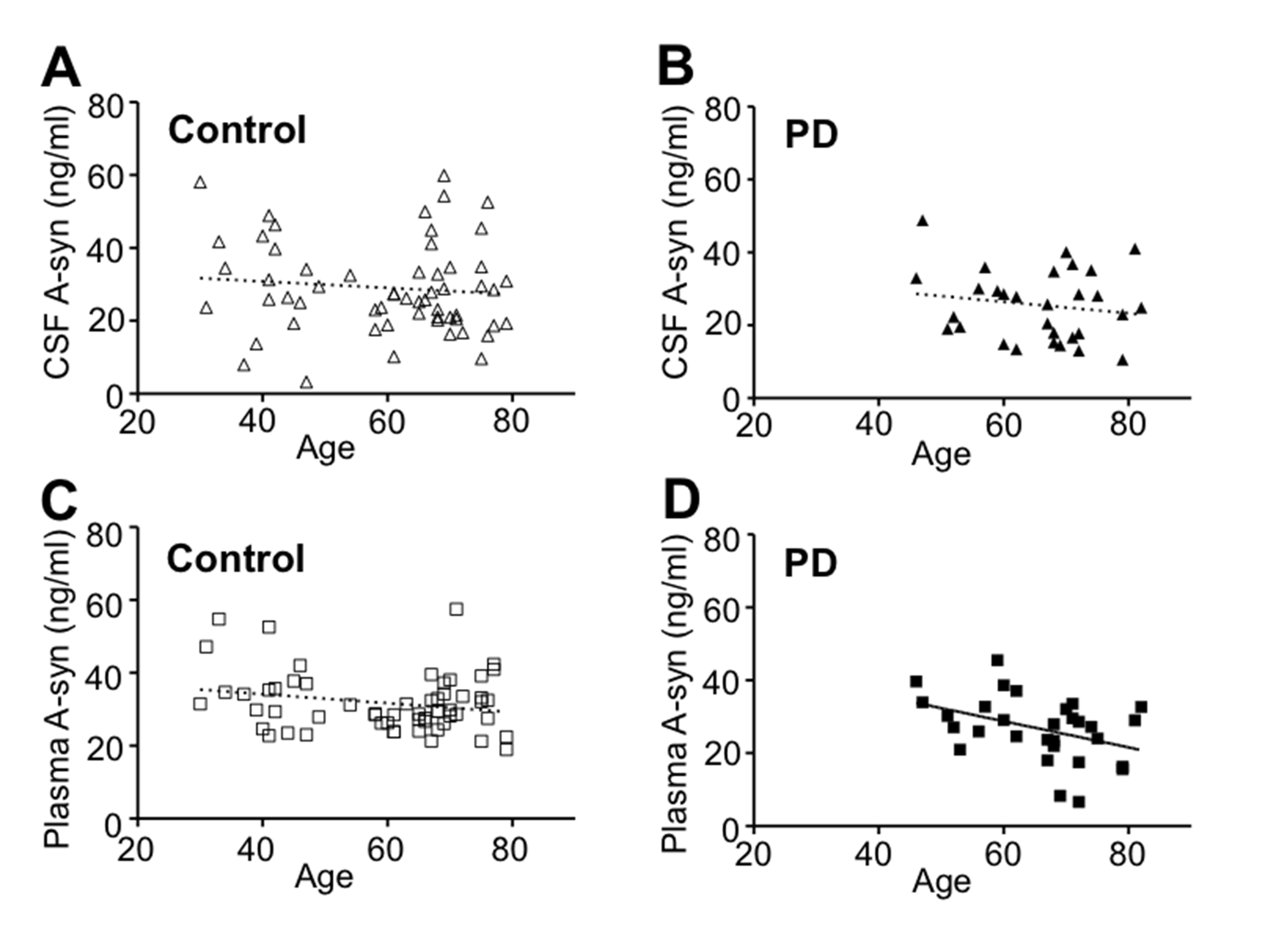

Supplement: S2 Fig — (A, B) Scatter plots of the age of the subjects versus CSF A-syn levels in the control (A) and PD (B) groups. (C, D) Scatter plots of the age of the subjects versus plasma A-syn levels in the control (C) and PD (D) groups. The lines in the graphs represent regression lines, with solid and dashed lines representing significant and non-significant correlations, respectively. Regression analyses revealed a non-significant correlation between the age of the controls and CSF A-syn levels (A; p = 0.46) or plasma A-syn levels (C; p = 0.20), as well as between the age of the PD patients and CSF A-syn levels (B; p = 0.40). The correlation between the age of the PD patients and the levels of plasma A-syn was significant (D; p = 0.03). (TIF) [file pone.0123162.s002.tif]
